# Current status and geographic inequities in solid organ and corneal transplantation in Ecuador (2010–2022)

**DOI:** 10.7717/peerj.21282

**Published:** 2026-06-04

**Authors:** Daniel Romero-Alvarez, Erick Velasteguí-Peñafiel, María Esther Castillo, Felipe Ortiz, Héctor Gutiérrez-Espinoza, Isabel Baroja, Carlos Bastidas-Caldes

**Affiliations:** 1 Instituto Nacional de Biodiversidad (INABIO), Quito, Ecuador; 2 Research Group of Emerging and Neglected Diseases, Ecoepidemiology and Biodiversity, Health Science Faculty, School of Biomedical Sciences, Universidad Internacional SEK, Quito, Ecuador; 3 Faculty of Medicine, Biomedical Sciences, Graduate School, Universidad de Chile, Santiago, Chile; 4 National Institute for Organ, Tissue, and Cell Donation and Transplantation (INDOT), Quito, Ecuador; 5 Faculty of Education, Universidad Autónoma de Chile, Santiago, Chile; 6 IDIBAPS Biomedical Research Institute, Universidad de Barcelona, Barcelona, Spain; 7 School of Medicine, Universidad de Especialidades Espíritu Santo, Samborondón, Ecuador

**Keywords:** Organ, Tissue, Urban, Rural, Waiting list, Transplantation, Transplants, Ecuador

## Abstract

**Background:**

Organ and tissue transplantation is an essential therapy for end-stage disease. However, in Ecuador, systematic evidence on waiting list dynamics, demographic and clinical patterns, and equity in access remains scarce. The absence of comprehensive analyses has limited the capacity to identify disparities and to inform evidence-based allocation policies.

**Objective:**

This study aimed to characterize the national transplant waiting list in Ecuador from 2010 to 2022, to assess demographic, clinical, and geographic disparities to provide insights into donor–recipient compatibility and equity in allocation.

**Study Design:**

We conducted a retrospective cohort study using data from the Ecuadorian National Institute for Organ and Tissue Donation and Transplantation (INDOT). The dataset included all patients listed for kidney, liver, heart, lung, and corneal transplants between 2010 and 2022. Variables analyzed included age, sex, comorbidities, geographic origin, and mortality while on the waiting list. Descriptive statistics were used to characterize the population and compare trends across organ types.

**Results:**

A total of 6,523 individuals were listed during the study period: 59% for corneal, 35.7% for kidney, 4.9% for liver, 0.3% for heart, and 0.06% for lung transplantation. Men predominated in corneal (72.3%) and heart (72.7%) lists, whereas women predominated in kidney (61%) and liver (57%) lists. Overall mortality while on the waiting list reached 21.3%. Geographic disparities were evident, with most transplant programs concentrated in Quito, Guayaquil, and Cuenca.

**Conclusions:**

This nationwide study demonstrates persistent inequities in Ecuador’s transplant system reflected in geographic concentration, sex-specific trends, and mortality among kidney and liver candidates. Findings highlight the need to expand programs beyond major cities, strengthen donor identification strategies, and integrate clinical, socioeconomic, and genetic factors into allocation policies.

## Introduction

Chronic diseases usually lead to irreversible organ damage. The increasing prevalence of unhealthy lifestyles driven by socio-economic and environmental inequalities such as poverty, housing and food insecurity, and systemic violence have increased chronic disease incidence posing a significant medical challenge worldwide (Lago et al., 2018). In response, organs, tissue, and cell transplantation have become well-established and often essential treatments to ensure long-term survival (Matesanz et al., 2009). The demand for transplants consistently exceeds organ availability, highlighting a critical gap in healthcare systems (Piao et al., 2018).

According to the Global Observatory on Donation and Transplantation, organ replacement therapy distribution in 2020 was as follows: kidney (62%), liver (25%), heart (6%), lung (5%), and pancreas (2%), accounting for a total of 129,681 transplant surgeries worldwide (Global Observatory on Donation Transplantation, 2021). By 2021, this number increased to 144,302, reflecting an approximate 11% growth, attributed to continuous improvements in organ procurement and the efficiency of transplant systems (Churchill & Gudgel, 2025). Additionally, the American Academy of Ophthalmology estimated that nearly 185,000 corneal transplants are performed annually in 166 countries (Gain et al., 2016).

Despite global progress, Ecuador faces significant structural challenges in achieving international transplantation standards. In 2022, the country performed a total of 597 transplants, distributed as follows: solid organs (32%), corneal tissues (56%), and hematopoietic progenitor cells (12%) (Espinel Lalama, Castillo Muñoz & Espín Vaca, 2023). The national organ donation rate in 2020 was 3.8 per million population (pmp), considerably lower than those of neighboring countries such as Colombia (15.9 pmp), Argentina (29.5 pmp), or Brazil (34.9 pmp) (Matesanz et al., 2009). The Ecuadorian lower rate of organ transplantation might be explained due to shortages in medical resources, bureaucratic obstacles for interventions of private entities, and limited coordination with scientific societies, as highlighted in a time series study of kidney transplants in Ecuador from 1976 to 2022 (Jiménez & Jiménez, 2022).

Although Ecuador has observed an increase in organ donors since 2009 due to the establishment of the National Institute for Organ Donation and Transplantation (INDOT, in Spanish) and the implementation of a legal framework aimed at enhancing efficiency and transparency in organ allocation, the persistent low transplantation rate underscores the importance to strengthen the national health system and expand donation programs to meet the growing transplant demand (Ministerio de Salud Pública de Ecuador, 2011; Espinel Lalama, Castillo Muñoz & Espín Vaca, 2023).

The current study describes the clinical and sociodemographic variables of patients who are on the waiting list for transplant between 2010 to 2022 in Ecuador. Our study builds upon the work of Espinel Lalama, Castillo Muñoz & Espín Vaca (2023), by expanding the study period, incorporating additional variables, and depicting the temporal and geographic distribution of transplant demand and organ supply for four organs (kidney, liver, heart, and lung) and one tissue (cornea) in Ecuador.

## Methods

### Design/ethics

The present study was conducted in accordance with the Strengthening the Reporting of Observational Studies in Epidemiology (STROBE) guidelines for retrospective studies (Von Elm et al., 2008). The research protocol (Universidad de las Américas Ethics committee ID: 2023-EXC-0098) was reviewed by Ministry of Public Health accredited Institutional Review Board and was classified as exempt from full ethical review and from obtaining individual informed consent. The study used secondary, anonymized data from the national Ecuadorian organ-transplant waiting list provided by the INDOT. The database had been previously coded and anonymized by INDOT personnel to eliminate all personally identifiable information. All investigators involved in this study signed data-confidentiality agreements with INDOT.

### Patients

Ecuador is a middle-income country at northwestern South America with a population of ∼17 million according to the official 2022 census (Instituto Nacional de Estadística y Censos, 2024). Although a multi-ethnical country, up to 77.5% of its population is mestizo, a colonial legacy when Europeans and Indigenous mixed their ancestry. Approximately 36.1% of its population lives in locales identified as rural (Torres et al., 2025). Ecuador’s health system is fragmented with the Ministry of Public Health (MoH) providing services to the uninsured, the social security system providing services to the publicly insured based on their voluntary or employer payments, and the private sector services paid out-of-pocket, by private insurers, or by the public sector based on agreements with the MoH and the social security system (Torres & López-Cevallos, 2018). This study included data from patients who were on the national Ecuadorian waiting list for transplant of four organs (kidney, liver, heart, and lung) and one tissue (cornea) in Ecuador between 2010 and 2022 (Velasteguí et al., 2025). All raw demographic and clinical data are available in Table S1.

We categorized patients in two groups: those for organ and those for corneal transplantation as the intervention and logistics for an organ transplant differ vastly from a corneal one. For example, corneal transplants can waive histocompatibility exams as the tissue is avascular. Conversely, organ transplants require far more testing to minimize the risk of graft rejection (Oli et al., 2022). In Ecuador, The Organic Law on Donation and Transplantation of Organs, Tissues, and Cells (Asamblea Nacional de la República del Ecuador, 2011) establishes that organs and tissues are obtained from brain-dead donors. Corneal procurement is permitted from donors following cardiac arrest due to high sanitary demand. While the allocation of organs and corneas is strictly governed by the national single waiting list, other tissues are centralized and distributed by the National Tissue Bank (BANTEC) to authorized healthcare facilities according to their specific requirements (Asamblea Nacional de la República del Ecuador, 2011).

### Outcomes measures

For all patients included in the national Ecuadorian transplant waiting list, sociodemographic variables such as age, sex, and geographic location, along with clinical variables such as organ requested, waiting list status, transplant date, number of transplants, and number of deceased patients, were collected.

### Statistical analysis

Descriptive statistics were used for patients’ sociodemographic and clinical variables. Categorical variables are presented as counts and percentages (%). Statistical analyses were performed using Stata 16.0 (Stata; College Station, TX, USA) and R (R Core Team, 2023) *via* R-studio (https://rstudio-education.github.io/hopr/starting.html). Incidence of transplant demand and recipients was calculated using the counts of each category as numerator and the total population of Ecuador according to the 2022 official census (https://www.censoecuador.gob.ec/) at the second administrative country unit, *i.e.,* cantons, as denominator. Rural cantons were classified as such if more than 60% of the population lived in rural parishes (Table S2; Supplementary Information 1). Figures and maps were generated using Biorender, R Studio, and QGIS.

## Results

**Figure 1:**
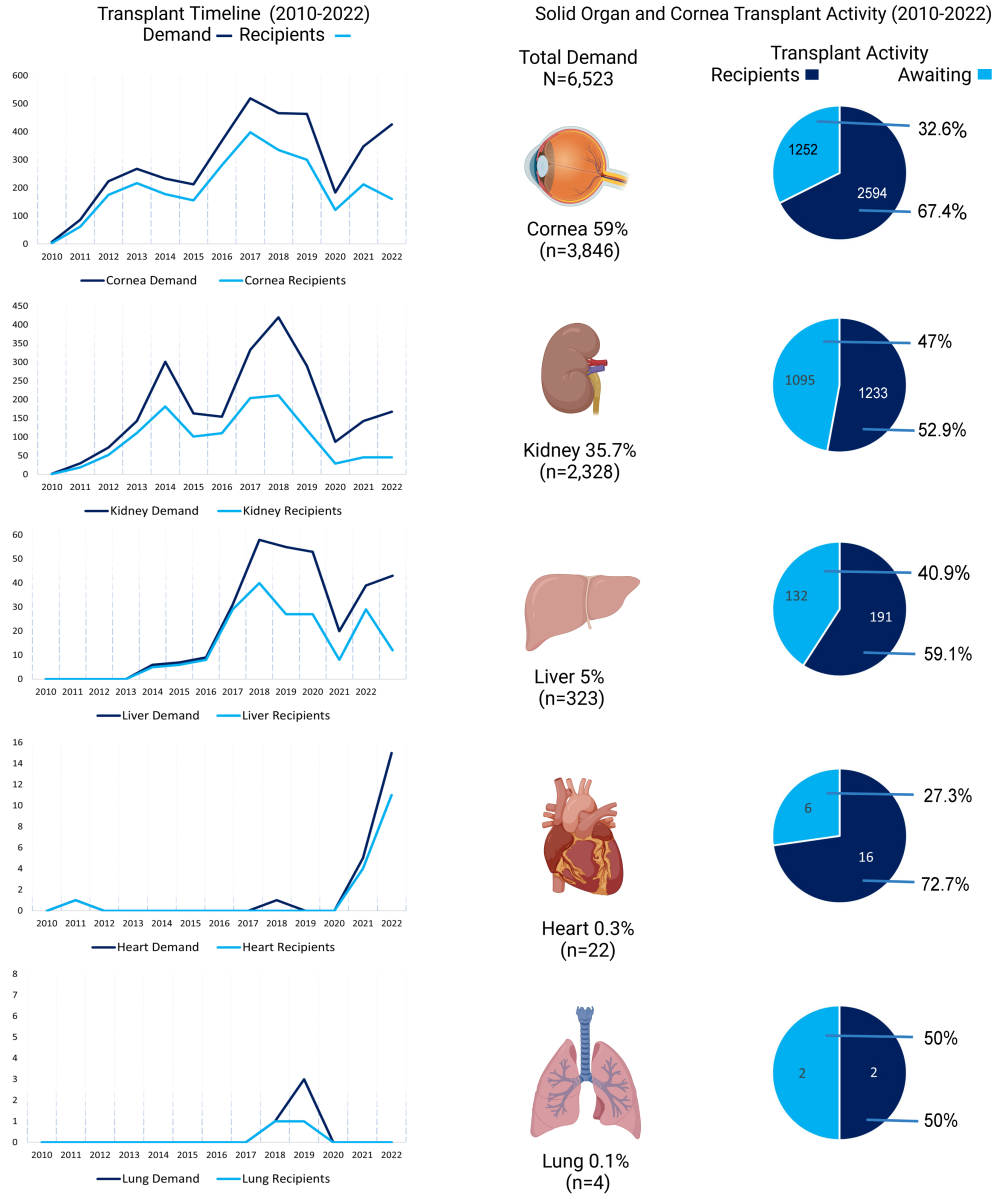
Trends and distribution of organ and corneal transplants in Ecuador (2010–2022). The left panel illustrates the temporal trends in transplant demand and the number of recipients for cornea, kidney, liver, heart, and lung transplants. The right panel summarizes transplant activity, with pie charts depicting the proportion of individuals who successfully received a transplant *versus* those who remained on the waiting list or passed away. Percentages and total numbers for each organ type are also indicated.

Between 2010 to 2022, 6,523 individuals were registered in the Ecuador transplant waiting list. Among them, 59% (*n* = 3,846) were listed for corneal transplants, 35.7% (*n* = 2,328) for kidney, 4.9% (*n* = 323) for liver, 0.3% (*n* = 22) for heart, and 0.06% (*n* = 4) for lung transplants (Fig. 1). The proportion of patients who underwent a transplant varied depending on the organ requested: 52.5% for kidney, 58.5% for liver, 72.7% for heart, and 50% for lung transplants. Up to 62.7% of patients listed received a corneal transplant (Fig. 1).

### Demographic and geographic distribution of transplant demand and mortality

A total of 90.3% of patients resided in urban areas listed in the analyzed database of transplant donors and recipients, conversely, 9.7% lived in rural regions (Table 1). The highest demand for transplants was recorded between 2016 and 2018, followed by a sharp decline in 2020, coinciding with the COVID-19 pandemic, which significantly impacted transplantation procedures and monitoring activities (Fig. 1, Table 1).

**Table 1:** Sociodemographic information of patients on the Ecuadorian transplant list 2010-2022. Values are presented as counts and percentages shown in parentheses. Percentages in organ/tissue columns are calculated using organ or tissue-specific waiting list totals as denominators (cornea *N* = 3,846; kidney *N* = 2,328; liver *N* = 323; heart *N* = 22; lung *N* = 4). Percentages in the Total column are calculated using the overall study population as the denominator (*N* = 6,523). Previous transplants refer to any solid organ or tissue transplant received before registration on the current waiting list and do not necessarily correspond to the same organ. ^∗^Corneal transplantation was excluded from mortality analyses; denominators differ accordingly for dead on the waiting list or after transplant.

**Characteristic**	**Total N (%)**	**Cornea (%)**	**Kidney (%)**	**Liver (%)**	**Heart (%)**	**Lung (%)**	
Total	6,523 (100.0)	3,846 (59.0)	2,328 (35.7)	323 (4.9)	22 (0.3)	4 (0.06)	
**Sex**							
Male	3,846 (59.0)	2,781 (72.3)	909 (39.0)	139 (43.0)	16 (72.7)	1 (25.0)	
Female	2,677 (41.0)	1,065 (27.7)	1,419 (61.0)	184 (57.0)	6 (27.3)	3 (75.0)	
**Age (years)**							
Child (0–5)	14 (0.2)	13 (0.3)	1 (0.0)	0 (0.0)	0 (0.0)	0 (0.0)	
Scholar child (6–11)	108 (1.7)	73 (1.9)	33 (1.4)	2 (0.6)	0 (0.0)	0 (0.0)	
Teenager (12–19)	600 (9.2)	472 (12.3)	116 (5.0)	12 (3.7)	0 (0.0)	0 (0.0)	
Young adult (20–39)	2,224 (34.1)	1,304 (33.9)	868 (37.3)	43 (13.3)	6 (27.3)	1 (25.0)	
Adult (40–59)	2,002 (30.7)	840 (21.8)	971 (41.7)	174 (53.9)	13 (59.1)	3 (75.0)	
Elderly (>60)	1,575 (24.1)	1,144 (29.7)	339 (14.6)	92 (28.5)	3 (13.6)	0 (0.0)	
**Waiting time (months)**							
0–12	2,992 (45.9)	2,064 (53.7)	734 (31.5)	176 (54.5)	16 (72.7)	2 (50.0)	
13–24	458 (7.0)	237 (6.2)	210 (9.0)	11 (3.4)	0 (0.0)	0 (0.0)	
25–36	246 (3.8)	121 (3.1)	121 (5.2)	4 (1.2)	0 (0.0)	0 (0.0)	
37–48	148 (2.3)	66 (1.7)	81 (3.5)	1 (0.3)	0 (0.0)	0 (0.0)	
49–60	76 (1.2)	35 (0.9)	41 (1.8)	0 (0.0)	0 (0.0)	0 (0.0)	
>60	89 (1.4)	34 (0.9)	55 (2.4)	0 (0.0)	0 (0.0)	0 (0.0)	
Still waiting	2,487 (38.1)	1,252 (32.6)	1,084 (46.6)	131 (40.6)	6 (27.3)	2 (50.0)	
No data	27 (0.4)	37 (1.0)	2(0.1)	0	0	0	
**History of transplantation (Number of prior transplants)**							
0	2,487 (38.1)	1,252 (32.6)	1,095 (47.0)	132 (40.9)	6 (27.3)	2 (50.0)	
1	3,842 (58.9)	2,411 (62.7)	1,223 (52.5)	189 (58.5)	16 (72.7)	2 (50.0)	
2	173 (2.7)	162 (4.2)	10 (0.4)	2 (0.6)	0 (0.0)	0 (0.0)	
3	17 (0.3)	17 (0.4)	0 (0.0)	0 (0.0)	0 (0.0)	0 (0.0)	
4	3 (0.0)	3 (0.1)	0 (0.0)	0 (0.0)	0 (0.0)	0 (0.0)	
5	1 (0.0)	1 (0.0)	0 (0.0)	0 (0.0)	0 (0.0)	0 (0.0)	
**Locality**							
Urban	5,890 (90.3)	3,416 (88.9)	2,142 (92.0)	305 (94.4)	22 (100.0)	3 (75.0)	
Rural	633 (9.7)	430 (11.2)	186 (7.9)	18 (5.6)	0 (0.0)	1 (25.0)	
**Deceased patients**							
Dead on waiting list	263/1235^∗^ (21.3)	NA	211/1095(19.3)	50/132 (37.9)	1/6(16.6)	1/2(50)	
Dead after transplant	3/1440^∗^ (0.1)	NA	1/1231 (0.08)	2/191(0.6)	0 (0.0)	0 (0.0)	

Analysis of sex distribution revealed distinct trends: men were more frequently listed for corneal (72.3%) and heart (72.7%) transplants, while women represented the majority in kidney (61%), liver (57%), and lung (75%) transplantation programs (Table 1).

During the study period (*i.e.,* 2010 to 2022), 21.3% of patients (n=263/1,235) died while waiting for a solid organ transplant in Ecuador (Table 1). The proportion of deaths varied across organ or tissue categories, with the highest observed for liver transplant candidates (37.9%, *n* = 50/132), followed by kidney transplant candidates (19.3%, *n* = 211/1,095). Mortality among heart and lung transplant candidates was 16.6% (*n* = 1/6) and 50% (*n* = 1/2) respectively, which should be interpreted cautiously considering the overall small number of patients in these latter groups. Post-transplant mortality was considerably lower, with only 0.1% of patients (*n* = 3/1,440) dying after receiving a transplant; this mortality was observed for patients receiving kidney (*n* = 1) and liver (*n* = 2) transplants (Table 1).

### Corneal tissue transplant population

**Figure 2:**
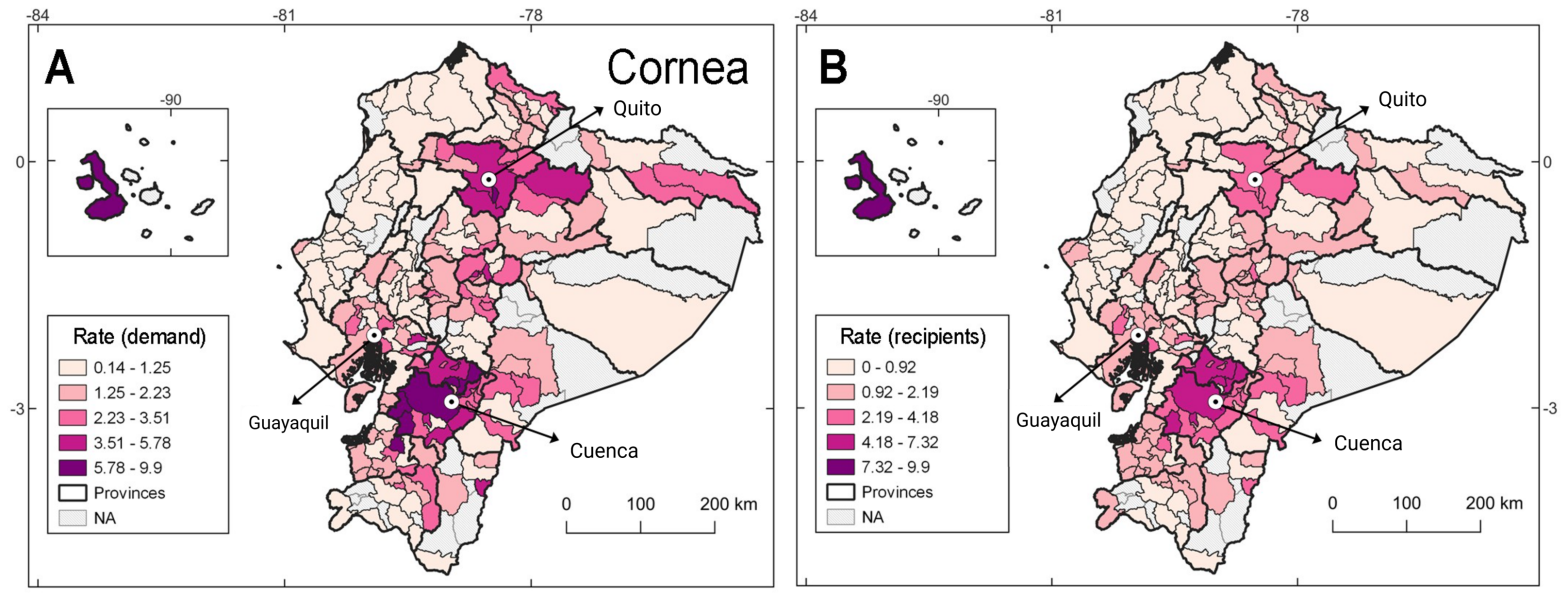
Geographic and demographic distribution of transplant demand and recipients for corneal tissue in Ecuador (2010–2022). (A) Depicts the geographic distribution of patients on transplant waiting list while (B) illustrates the geographic distribution of transplant recipients. These data provide insights into regional disparities in organ availability and transplantation rates.

A total of 53.7% of patients waiting for corneal transplants underwent their procedure within the first 12 months. Individuals awaiting corneal transplants are distributed nationwide (Fig. 2; Table S2). Corneas are the most requested and transplanted tissue in Ecuador meeting 59% of the overall transplant demand (Table 1). Patients referred for cornea tissue transplants were mainly from northern and southern Andean region, with a larger demand from the southern province of Azuay which was also the province that reported the majority of actual transplants (Fig. 2; Table S2).

### Solid organ transplant population

**Figure 3:**
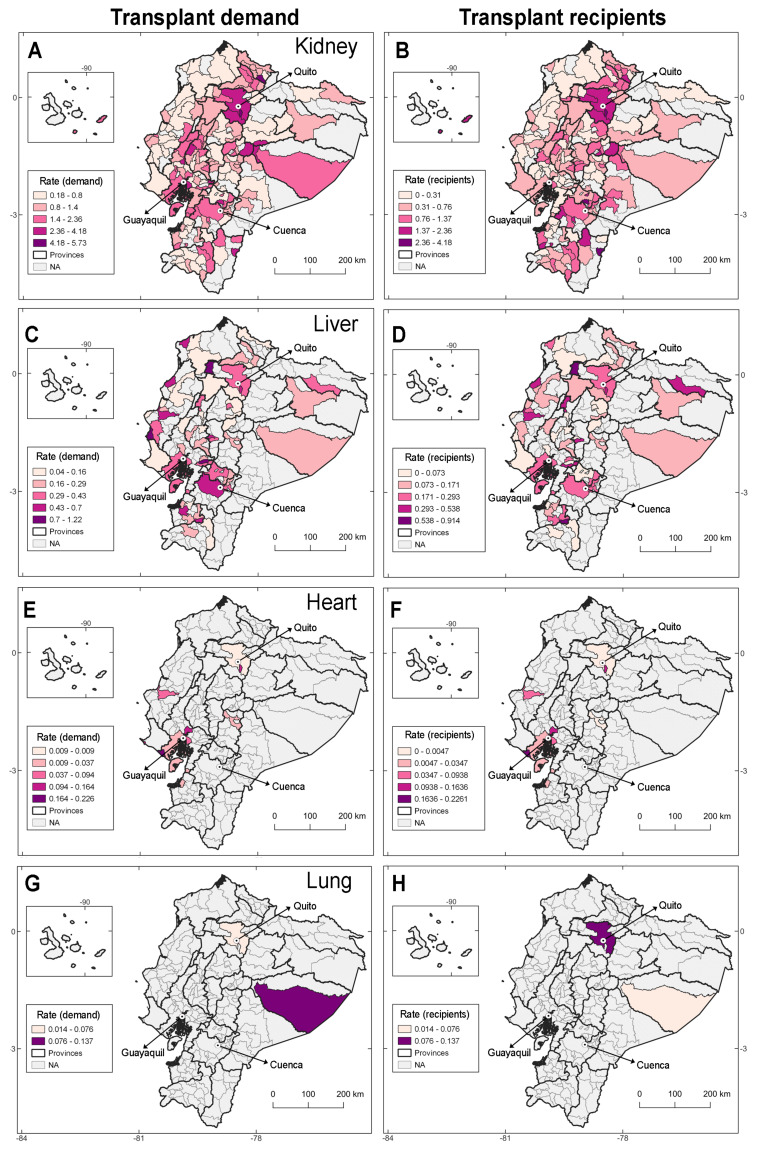
Geographic and demographic distribution of transplant demand and recipients for solid organs in Ecuador (2010–2022). Left panels depict the geographic distribution of patients on transplant waiting list while the right panels illustrate the geographic distribution of transplant recipients. These data provide insights into regional disparities in organ availability and transplantation rates.

For solid organs, time-to-transplant varied. Within the first year, 31.5% of kidney patients, 54.5% of liver patients, 72.7% of heart patients, and 50% of lung patients received their grafts (Fig. 1; Table 1). The demand for kidney transplants is concentrated mainly in Quito, Pichincha province; most recipients are from the same locale (Fig. 3; Table S2). Individuals awaiting liver transplants are sparsely distributed across Ecuador with important focus in the provinces of Pichincha, Azuay, and Guayas (Fig. 3). Demand for hearts (*n* = 22) and lungs (*n* = 4) is scarce in the country with actual transplants occurring for patients from urban cantons (Table 1, Fig. 3).

## Discussion

Our analysis demonstrates comprehensive trends in organ and corneal transplantation in Ecuador from 2010 to 2022, revealing a gap between transplant demand and organ availability, geographic disparities, and the impact of the COVID-19 pandemic on transplant activity. Notably, corneal transplants represented the highest proportion of registrations (59%), followed by kidney transplants (35.7%). Furthermore, we observed an overall mortality rate in the waiting list of 21.3% and that Cuenca canton in the province of Azuay concentrates the larger demand for corneal and liver transplants. Our findings highlight critical areas for intervention to improve accessibility and transplant outcomes.

Ecuador’s transplant programs are concentrated in three major urban centers—Quito, Guayaquil, and Cuenca—which house all accredited heart, liver, kidney, and corneal programs (Espinel Lalama, Castillo Muñoz & Espín Vaca, 2023). This centralization reflects a structural imbalance in service availability, limiting access for patients from rural and remote provinces, and contributing to the persistent geographic disparities observed in our data. Accordingly, most transplant recipients are distributed in these three cantons (Figs. 2 and 3). Notably, heart transplants are reported only from urban centers (*i.e.,* 100%, Table 1).

The COVID-19 pandemic had a significant impact on Ecuador’s transplant activity, causing a sharp decline in heart, liver, kidney, and corneal transplant programs from 2020 onwards, with a partial recovery observed by 2022 (Fig. 1). This trend was also reported in other Latin American countries, such as Argentina (Ministerio de Salud Argentina, 2020) and Colombia (Martin, Sierra-Ruiz & Barrera-Lozano, 2020). However, beyond the pandemic, longstanding social, technical, and legal challenges—including limited intensive care unit (ICU) availability, logistical barriers in organ procurement, and inadequate distribution networks—have contributed to systemic vulnerabilities in the transplant system (Bermeo, Ostos & Cubillos, 2009).

Evidence from international settings shows that inefficiencies in organ procurement organizations, delays in transport logistics, and suboptimal allocation mechanisms continue to result in missed opportunities for transplantation, often leaving viable organs unused (Olawade et al., 2025). Likewise, experiences in regions with limited infrastructure emphasize that insufficient transplant centers, restricted critical care capacity, and fragmented governance significantly hinder program expansion and exacerbate geographic inequities (Kute et al., 2025). These persistent weaknesses indicate that, in Ecuador as elsewhere, the sustainability of transplantation requires recovery from the pandemic’s disruption and the structural strengthening of infrastructure, coordination, and legal frameworks.

Although Ecuador has made significant progress in organ transplantation since 2007, performing 7,373 transplants up to 2022. In that year alone, 597 organs, tissue, and cell transplants were conducted, raising the national donation rate to 5.30 pmp (Espinel Lalama, Castillo Muñoz & Espín Vaca, 2023). However, the country faces significant disparities in access to transplantation, particularly for kidney transplants, where only 4% of patients on dialysis are included in the waiting list. Chronic kidney disease is a major public health issue in Ecuador, with annual treatment costs reaching approximately 650 million USD (Torres et al., 2022; Espinel Lalama, Castillo Muñoz & Espín Vaca, 2023).

### Demographic and geographic trends in transplant demand

The distribution of transplant demand is heterogenous across Ecuador. Urban areas account for 90.29% of patients on the waiting list, whereas only 9.70% reside in rural regions (Table 1). Limited access to specialized healthcare services may explain this disparity, as patients in rural areas face challenges in joining transplant waiting lists. It might be the case that a lack of understanding of the legal framework and logistics involved for a successful transplant prevent health providers in rural areas of Ecuador to recommend this treatment option and successfully derive patients to the transplant waiting list. Answering this question escapes the scope of the present study and the available data (Table S1), however, improving health practitioners’ knowledge on transplant practices and infrastructure might increase public participation on transplant programs (Alex et al., 2019).

Moreover, rural areas are usually inhabited by people in socioeconomic hardships, which have been related to disparities on health accessibility that directly determine their participation in transplant programs (Jaure et al., 2025; Neuberger, 2026). People living in rural areas are also those that belong to minority ethnic groups that are systematically less likely to be in a transplant waiting list as shown, for example, in the United States (US) for Black and Hispanic populations (Clark-Cutaia et al., 2024). Although an ethnic analysis of those in the transplant waiting list in Ecuador was beyond this study, it might be the case that indigenous communities are largely underrepresented as they are in an overall disadvantage in comparison with other Ecuadorian groups, showing larger malnutrition levels and higher mortality (Torres et al., 2025; Masala & Monni, 2017; Amano et al., 2021; Cuéllar et al., 2022; Bastidas-Caldes et al., 2024). Future studies can aim to understand these disparities to improve the accessibility to transplant programs in Ecuador.

Between 2010 and 2022, 21.3% of Ecuadorian patients (*n* = 263/1,235 individuals) on the waiting list died before receiving a transplant (Table 1). Conversely, mortality among transplants recipients was 0.1% (*n* = 3/1,440) underscoring critical deficiencies in organ availability and waiting time management, but adequate post-transplant survival times (Table 1). In comparison, early-decade data from the US reported an annual mortality rate of 6.3 per 100 patient-years for patients on dialysis awaiting kidney transplantation (Wolfe et al., 1999), a figure that has since declined despite increasing incidence of end-stage renal disease (Hernández et al., 2018). Similarly, in 2014, studies in the US showed that liver waitlist mortality varied significantly by region, ranging from 1.6% in areas with shorter waiting times to 8.4% in regions with prolonged waiting times (Goyes et al., 2021). While cross-country comparisons should be interpreted cautiously due to socioeconomic and healthcare system differences, the findings of our study demonstrate that shortening waiting times for transplants should be considered a paramount target to decrease mortality (Jiménez & Jiménez, 2022).

### Organ-specific considerations

#### Corneal tissue transplantation

Corneal pathology is considered the fifth leading cause of blindness worldwide (Mathews et al., 2018). Despite the global shortage of corneal tissue (Gain et al., 2016), our data showed that between 2010–2022 Ecuador was able to successfully transplant 2,594 corneas which represent the 67.4% of the overall demand (Fig. 1). As in other countries, corneas where the most available source for transplantation in Ecuador (Table 1), however, due to limitations of our database, we were unable to discriminate whether cornea donors were also donors for solid organs. Allen et al. (2025) showed that there are more multi-organ donors than actual eye-donors and explained these differences in relation to cultural beliefs and fear of disfigurement of the deceased individual. As this study was performed in New Zealand, a focused study in our region might reveal a dissimilar pattern as, theoretically, modern commitment for eye donation might have improved (Williams & Muir, 2018).

Main etiologies causing corneal damage include Fuchs dystrophy in the elderly and keratoconus in younger populations (Gain et al., 2016). Although we were unable to discriminate the etiologies for transplants in our study, young adults (20–39 years) and the elderly (>60 years) were the categories with most patients in the list for corneal transplants (Table 1). Future studies should answer questions related with etiologies for transplants in Ecuador as they might unveil risk factors for targeted prevention campaigns. Specifically, the geographic demand of corneal transplants in Ecuador is concentrated across the Andean region, with the largest rate of demand from the province of Azuay at the South of the country (Fig. 2). The city of Cuenca in Azuay is the third most populated Ecuadorian city and, as such, is an important enclave of health infrastructure, potentially explaining why 88.9% of corneal transplants were done in urban populations (Table 1). In the city of Bogota, Colombia, 78.7% *versus* 13.4% of urban and rural populations, respectively, were part of the corneal transplant donor list between 2010–2017; with an overall male majority (56.8%; Mora et al., 2021), like the Ecuadorian findings. Thus, it is likely that across South America, knowledge of transplant pipelines for corneal grafts and improvements on health centers availability might overcome the gap between urban and rural populations on corneal transplants, as well as for solid organs.

#### Kidney transplantation

Women represent 61% of kidney transplant candidates in Ecuador, with 37.3% aged 20–39 years and 41.7% aged 40–59 years. This aligns with reports indicating a higher prevalence of diabetes, systemic lupus erythematosus, and glomerulonephritis—conditions that contribute to kidney failure—among women (Chaudry et al., 2017; Maric-Bilkan, 2020). However, only 4% of dialysis patients nationwide are listed for kidney transplantation (Jiménez & Jiménez, 2022), suggesting substantial systemic barriers to referral and evaluation that may distort the apparent sex distribution of candidates. Previous studies have underscored that chronic kidney disease constitutes a significant epidemiological and socioeconomic challenge for Ecuador, given its high incidence and associated costs, within a context of economic crisis (Torres et al., 2022; Espinel Lalama, Castillo Muñoz & Espín Vaca, 2023). Consistently, kidney demand is reported across the country although the larger records for kidney recipients are concentrated in Quito, the capital urban center of the country in Pichincha province (Fig. 2). Addressing these issues requires the expansion of the number of transplant programs and improving early referral pathways, particularly for underserved populations.

#### Liver transplantation

The findings of this study indicate that 57% of liver transplant candidates in Ecuador are women, primarily aged 40–59 years, which aligns with the higher prevalence of autoimmune hepatitis and primary biliary cirrhosis in female patients—both major indications for liver transplantation (Guy & Peters, 2013; Mínguez Sabater et al., 2022). However, the higher mortality rate from chronic liver disease and cirrhosis in men suggests sex-based differences in disease progression and access to transplantation (Jadlowiec & Taner, 2016; Trotter, 2017). The geographic concentration of liver transplant demand in sparse cantons of the Coast, and the southern urban center of Cuenca in the province of Azuay (Fig. 2) may reflect a combination of factors, from accessibility to health services, to actual pathology including higher obesity prevalence, dietary patterns, and possible heavy metal exposure, all of which require further investigation (Chan et al., 2015; Jadlowiec & Taner, 2016; Trotter, 2017; Mora-Verdugo et al., 2022).

#### Heart transplantation

In Ecuador, candidates for heart transplantation are predominantly male (72.7%), with most aged between 40 and 59 years. The concentration of the country’s two active heart programs in a single city, such as Quito, limits accessibility for patients in other regions. Beyond geographical inequities, the predominance of male candidates is strongly linked to the distribution of cardiovascular risk factors. Men tend to present at an earlier age with ischemic cardiomyopathy, largely driven by higher rates of behavioral risks such as smoking and alcohol consumption, along with biological traits like greater visceral adiposity and elevated serum creatinine levels, which accelerate progression to advanced heart failure (Kawamoto, Davis & Duvernoy, 2016; Rojas-Velázquez et al., 2020). In contrast, women often manifest coronary disease at older ages and with different phenotypes, such as microvascular dysfunction, hormonal influences, and a higher burden of hypertension (Kawamoto, Davis & Duvernoy, 2016; Rojas-Velázquez et al., 2020). These sex-specific differences have been consistently documented in cardiovascular and transplant literature, which highlights persistent male predominance among candidates and recipients, barriers in referral and allocation, and even differences in post-transplant outcomes (Awad, Shah & Griffith, 2022; Manla et al., 2022; Chung, Hartman & De Filippis, 2023; De Filippis et al., 2023). This trend is therefore expected to persist.

#### Lung transplantation

From 2010 to 2022, only four patients have been recorded as candidates for lung transplantation in Ecuador. From them, two patients successfully received lungs in the country between 2018 and 2019 (Fig. 1). Lower numbers in this category reflect the lack of an official program leading treatment for these patients and prevent us from highlighting any trend.

### Limitations of the study

As a retrospective analysis based on secondary data from the national transplant registry (INDOT), potential limitations include incomplete or inconsistent reporting, missing values, and the absence of post-transplant outcome data, which preclude evaluation of graft survival and long-term patient prognosis. Moreover, the lack of systematic integration of socioeconomic and healthcare access indicators—such as insurance coverage, education level, or distance to transplant centers—restricts the capacity to disentangle the underlying causes of observed disparities. Donor-related data were also limited, preventing robust comparisons between living and deceased donor transplants across organ types. Cross-country comparisons should be interpreted cautiously as heterogeneity in healthcare infrastructure, listing criteria, and allocation policies may introduce bias. Finally, future research should incorporate clinical, environmental, and genetic factors to fully elucidate the demographic and geographic patterns of transplant demand in Ecuador.

Our aim in the present study was to present an updated description of transplants in Ecuador binding together information from tissues and different organs. As corneal and liver transplants present a series of completely different logistical challenges, we avoided the application of inference-based statistical analysis as multivariate regressions. However, we believe that studies aiming to explain patterns described in this study can benefit the country for policy development considering regional contexts (Figs. 2 and 3). Moreover, due to the Ecuadorian fragmented health system—divided in private and public providers—and the limited governmental health budget (Romero-Alvarez, López-Cevallos & Torres, 2023), potential solutions for increasing the number of rural people receiving transplants or decreasing waiting times for transplants should be framed under a systemic improvement of health accessibility and social care (Lago et al., 2018), approaches that should be considered worldwide.

## Conclusions

This study reveals persistent inequities in Ecuador’s transplant system, with kidney and corneal transplants representing the highest demand, yet facing substantial barriers to access particularly for rural patients. Male predominance was observed among corneal and heart transplant candidates, while women constituted the majority of kidney and liver transplant candidates. The limited availability of heart, lung, and pancreas transplant programs, combined with the disruptive effects of the COVID-19 pandemic, underscores systemic vulnerabilities. Addressing these challenges will require strengthening donor identification and allocation processes, expanding transplant programs beyond major cities, and investing in regional healthcare infrastructure to ensure equitable access nationwide.

##  Supplemental Information

10.7717/peerj.21282/supp-1 Supplemental Information 1 Raw data Classification criteria for patients registered on the national transplant waiting list, including organ type, demographic characteristics, blood group, urgency status, and hospital accreditation. The variables and their corresponding categorical codes are provided for standardization and analysis.

10.7717/peerj.21282/supp-2 Supplemental Information 2 Data for mapping transplant demand and transplant recipient

10.7717/peerj.21282/supp-3 Supplemental Information 3 Provinces of Ecuador Ecuador is divided into 24 administrative units called provinces. We overlay Ecuadorian provinces over an elevation map to depict the three ecoregions of the country determined by the presence of the Andes Mountain Ridge. The Coast is located west to the Andes, and the Amazon is located East to the Andes. Ecuador has an insular region, the Galapagos archipelago, which lies 1,600 km into the Pacific Ocean.

## Additional Information and Declarations

### Data Availability

The following information was supplied regarding data availability: The raw datasets are available in the Supplemental Files.
