# Supplementary material for: Current status and geographic inequities in solid organ and corneal transplantation in Ecuador (2010–2022)
Source: PeerJ. 2026 Jun 4;14:e21282. doi: 10.7717/peerj.21282 (PMC13242743; doi:10.7717/peerj.21282)
Supplement: Supplemental Information 3 — Ecuador is divided into 24 administrative units called provinces. We overlay Ecuadorian provinces over an elevation map to depict the three ecoregions of the country determined by the presence of the Andes Mountain Ridge. The Coast is located west to the Andes, and the Amazon is located East to the Andes. Ecuador has an insular region, the Galapagos archipelago, which lies 1,600 km into the Pacific Ocean. [file peerj-14-21282-s003.docx]

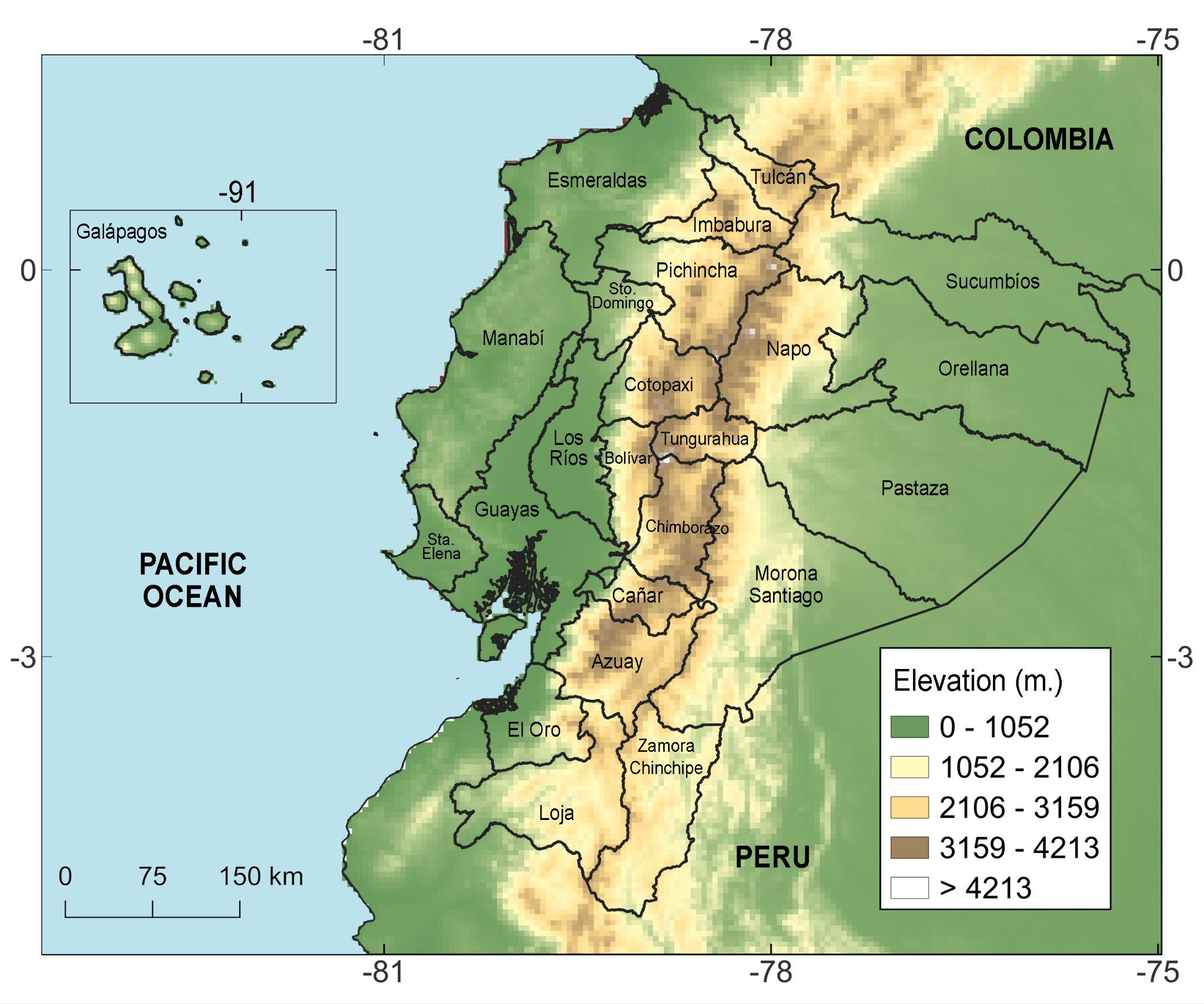


**Supplementary Figure 1. Provinces of Ecuador.** Ecuador is divided into 24 administrative units called provinces. We overlay Ecuadorian provinces over an elevation map to depict the three ecoregions of the country determined by the presence of the Andes Mountain Ridge. The Coast is located west to the Andes, and the Amazon is located East to the Andes. Ecuador has an insular region, the Galapagos archipelago, which lies ~1,600 km into the Pacific Ocean.
